# Plasma YKL-40 in the spectrum of neurodegenerative dementia

**DOI:** 10.1186/s12974-019-1531-3

**Published:** 2019-07-12

**Authors:** Anna Villar-Piqué, Matthias Schmitz, Peter Hermann, Stefan Goebel, Timothy Bunck, Daniela Varges, Isidre Ferrer, Joachim Riggert, Franc Llorens, Inga Zerr

**Affiliations:** 1 Department of Neurology, Clinical Dementia Center and National Reference Center for CJD Surveillance, University Medical School, Robert Koch 40, 37075 Göttingen, Germany; 2 German Center for Neurodegenerative Diseases (DZNE), Göttingen, Germany; 3 Network Center for Biomedical Research in Neurodegenerative Diseases, (CIBERNED), Institute Carlos III, Ministry of Health, Feixa Llarga s/n, L’Hospitalet de Llobregat, 08907 Barcelona, Spain; 4 Bellvitge Biomedical Research Institute (IDIBELL), Hospitalet de Llobregat, Spain; 5 Department of Pathology and Experimental Therapeutics, University of Barcelona, Hospitalet de Llobregat, Spain; 6 Department of Transfusion Medicine, University Medical School, Göttingen, Germany

**Keywords:** YKL-40, CHI3L1, Neurodegenerative dementia, Biomarker, Prion diseases, Plasma

## Abstract

**Background:**

Increased plasma YKL-40 has been reported in Alzheimer’s disease (AD), but its levels in other neurodegenerative diseases are unknown. Here, we aimed to investigate plasma YKL-40 in the spectrum of neurodegenerative dementias.

**Methods:**

YKL-40 was quantified in the plasma of 315 cases, including healthy controls (HC), neurological disease controls (ND), AD, vascular dementia (VaD), frontotemporal dementia (FTD), sporadic Creutzfeldt-Jakob disease (CJD) and Lewy body dementia (LBD). Diagnostic accuracy in the differential diagnostic context and influence of age and gender was assessed.

**Results:**

Highest YKL-40 levels were detected in CJD, followed by LBD, VaD, AD, FTD, ND and HC. YKL-40 was associated to age but not to sex. After controlling for age, YKL-40 was significantly elevated in CJD compared to HC (*p* < 0.001), ND, AD and VaD (*p* < 0.01) and in LBD compared to HC (*p* < 0.05). In CJD, YKL-40 concentrations were significantly higher at late disease stages.

**Conclusions:**

Plasma YKL-40 is significantly elevated in CJD regardless of clinical and genetic parameters, with moderate diagnostic accuracy in the discrimination from control cases. Our study discards a potential use of this biomarker in the differential diagnostic context but opens the possibility to be explored as a marker for CJD monitoring.

## Introduction

YKL-40, also known as chitinase-3-like protein 1 (CHI3L1), is a secreted glycoprotein expressed in several tissues and involved in activation of the innate immune system and in cell processes in relation to extracellular matrix remodeling [1–3]. Cerebrospinal fluid (CSF) concentrations of YKL-40 are significantly increased in sporadic Creutzfeldt-Jakob disease (CJD) and Alzheimer’s disease (AD), while other neurodegenerative dementias such as frontotemporal dementia (FTD), Lewy body dementia (LBD) and vascular dementia (VaD) show normal to slightly altered levels [4–6].

However, the potential role of YKL-40 as blood-based biomarker in the differential diagnostic has not been explored. In AD, plasma YKL-40 concentrations were reported to be increased, but with limited utility as a diagnostic marker [7] presenting moderate effect sizes according to meta-analysis studies [8]. The levels of plasma YKL-40 in other neurodegenerative dementias are unknown.

Here we evaluated the accuracy of plasma YKL-40 in the discrimination of neurodegenerative dementias from different etiologies.

## Methods

### Samples

Blood was collected in plasma-EDTA tubes at the Department of Transfusion Medicine (healthy controls (HC)) and at the Department of Neurology–Clinical Dementia Center (neurological disease controls (ND), sporadic Creutzfeldt-Jakob disease (CJD), Alzheimer’s disease (AD), frontotemporal dementia (FTD), Lewy body dementia (LBD) and vascular dementia (VaD)) in the Universitätsmedizin Göttingen (Germany) under same pre-analytical conditions.

The HC group was composed of healthy blood donors with absence of any relevant clinical finding. The ND group was composed of cases with neurological conditions not associated with neurodegenerative pathology diagnosed according to acknowledged standard neurologic clinical and para-clinical findings based on the International Classification of Diseases 10th Edition definitions. AD was diagnosed according to the National Institute on Aging-Alzheimer’s Association workgroups (NIA-AA) criteria [9]. CJD cases were classified as probable or definite according to diagnostic consensus criteria [10, 11]. The Lewy body dementia (LBD) group included dementia with Lewy bodies (DLB) and Parkinson’s disease dementia (PDD) cases. The diagnosis of DLB was based on the criteria of McKeith [12]. PDD diagnosis was based on the task force of the Movement Disorder Society (MDS) criteria [13]. FTLD was diagnosed according to the International Behavioural Variant FTD Criteria Consortium for bvFTD [14]. VaD diagnosis was based on clinical and radiological criteria as described by Roman (National Institute of Neurological and Communicative Disorders and Stroke and the Alzheimer’s Disease and Related Disorders Association (NINDS-AIREN)) [15]. In CJD cases, genetic testing of the codon 129 polymorphism of prion protein gene (*PRNP*) was conducted as described before [16]. For disease stage, samples were stratified in three categories according to whether they underwent blood uptake in the first (early) (time of blood uptake to disease onset/total duration of the disease < 0.33), second (middle) (0.33–0.66), or third (last) (> 0.66) stage of the disease. Disease duration was recorded as the time (in months) from symptom onset to the death of the patient.

### Plasma YKL-40 quantification

Plasma YKL-40 was measured with the MicroVue YKL-40 EIA ELISA kit from Quidel following the manufacturer’s instructions. Samples were diluted 1:2 to 1:4. Intra- and inter-assay coefficient of variation was 9% and 14%, respectively.

### Statistical analysis

We performed one-way analysis of variance followed by Tukey correction to compare age between disease groups. Association between YKL-40 levels and age was explored with Pearson correlation coefficient. We log-transformed YKL-40 concentration to obtain a normal distribution and applied analysis of covariance followed by Tukey correction to assess the differences between groups controlling for age as covariant. Association between YKL-40 and sex was investigated with the Mann-Whitney test. Receiver operating characteristic (ROC) curve and areas under the curve (AUC) with 95% confidence intervals (95%CI) were calculated to assess diagnostic accuracies between diagnostic groups. All analyses were performed using GraphPad Prism software and multcomp package in R [17].

## Results

**Fig. 1:**
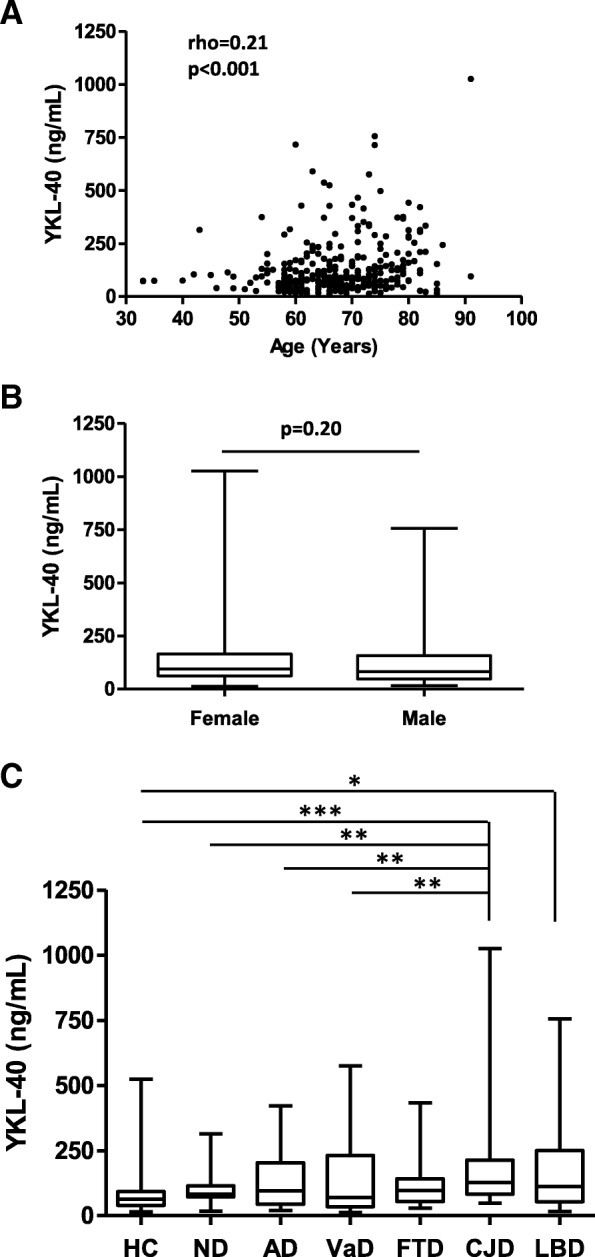
Plasma YKL-40 in the differential diagnostic context of neurodegenerative dementia and association with demographics. **a** Association between YKL-40 concentration and age at disease onset in the study population. **b** YKL-40 concentration stratified by sex. **c** YKL-40 concentration in HC, ND, AD, VaD, FTD, CJD and LBD. After age correction, YKL-40 was significantly elevated in CJD compared to HC (*p* < 0.001), ND, AD and VaD (*p* < 0.01) and in LBD compared to HC (*p* < 0.05). *p < 0.05, **p < 0.01 and ***p < 0.001

The study population consisted of 315 plasma age-matched cases with the exception of VaD cases that were significantly older than HC (*p* < 0.01) (Table 1). In the total population, YKL-40 was associated with age (rho = 0.21, *p* < 0.001) (Fig. 1a), but not with sex (*p* = 0.20) (Fig. 1b). Mean YKL-40 concentrations were higher in neurodegenerative dementias compared to HC (84 ng/mL) and ND (95 ng/mL), with highest concentrations detected in CJD (189 ng/mL), followed by LBD (167 ng/mL), VaD (140 ng/mL), AD (133 ng/mL) and FTD (125 ng/mL) (Table 1). After age correction, YKL-40 levels appeared significantly higher in CJD versus HC (*p* < 0.001), ND, AD and VaD (*p* < 0.01) and in LBD compared to HC (*p* < 0.05) (Fig. 1c). Area under the curve (AUC) for the discrimination of neurodegenerative dementia groups from controls ranged from 0.56 to 0.81 for HC and from 0.51 to 0.72 for ND, indicating low diagnostic value except in the discrimination between CJD from HC, which achieved a moderate value (0.81) (Table 1).

**Table 1:** Demographic, plasma YKL-40 concentrations and diagnostic accuracy in discrimination from healthy controls and neurological disease groups

	*n*	Sex (f/m)	Age (years)	YKL-40 (ng/mL)		AUC (95% CI)	
				Mean + SD	95% CI	vs. HC	vs. ND
HC	70	22/48	66 ± 5	84 ± 84	63–104		0.64 (0.53–0.74)
ND	44	26/18	66 ± 12	95 ± 61	76–114	0.64 (0.53–0.74)	
AD	50	25/25	69 ± 10	133 ± 110	102–164	0.62 (0.51–0.73)	0.55 (0.43–0.67)
VaD	22	8/14	72 ± 10**	140 ± 150	73–206	0.56 (0.40–0.72)	0.51 (0.34–0.68)
FTD	17	11/6	68 ± 12	125 ± 108	69–181	0.65 (0.50–0.80)	0.55 (0.38–0.73)
CJD	78	51/27	67 ± 8	189 ± 167	151–227	0.81 (0.74–0.88)	0.72 (0.63–0.81)
LBD	34	13/21	69 ± 8	167 ± 157	112–222	0.70 (0.59–0.81)	0.63 (0.49–0.76)

Number of cases (*n*), sex (female [f]/male [m]), age in years (mean values ± standard deviation (SD)), YKL-40 plasma concentrations (mean values ± SD) and 95% confidence interval (95% CI)) and area under the curve (AUC) with 95% CI values for each dementia diagnostic comparison versus HC and ND are indicated

**p < 0.01

**Fig. 2:**
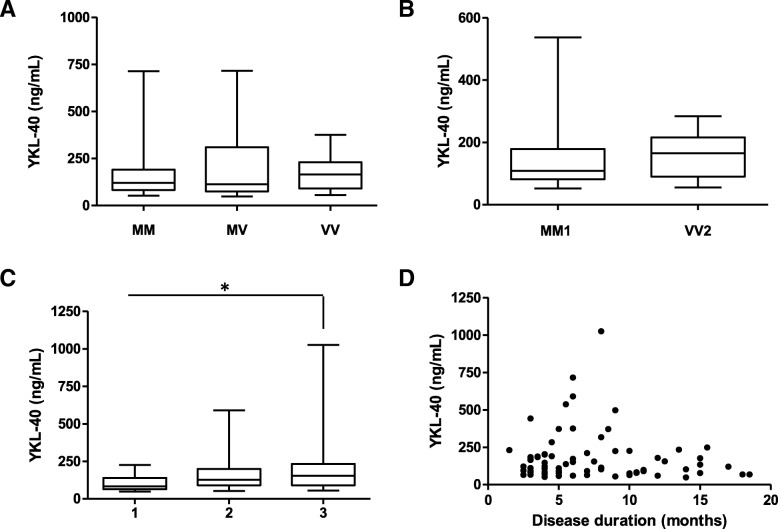
Influence of genetic and clinical data on plasma YKL-40 concentrations in CJD patients. **a** YKL-40 in CJD stratified by prion protein gene (*PRNP*) codon 129 polymorphism (M = methionine, V = valine). Kruskal-Wallis test followed by Dunn’s post hoc test (correction for multiple testing) was applied. **b** YKL-40 in CJD MM1 and VV2 subtypes. Mann-Whitney *U* test was used. **c** YKL.40 concentrations stratified by disease stage at the time of blood collection. Samples were grouped into three categories according to whether they underwent blood uptake in the first (< 0.33), second (0.33–0.66), or third (> 0.66) stage of the disease. Kruskal-Wallis test followed by Dunn’s post hoc test (correction for multiple testing) was applied, *p < 0.05. **d** Association between YKL-40 concentrations and disease duration (months) in CJD patients. Spearman rank correlation was used

Since the CJD group presented the most significant YKL-40 increase compared to other diagnostic groups, we conducted further investigations. We stratified CJD cases depending on the codon 129 polymorphism of *PRNP* gene, but no differences in YKL-40 concentrations were detected between methionine/methionine (MM) (165 ng/mL, *n* = 49), methionine/valine (MV) (212 ng/mL, *n* = 10) and valine/valine (VV) (173 ng/mL, *n* = 13) cases (Fig. 2a). Similarly, no differences were detected between the most prevalent CJD subtypes, MM1 (136 ng/mL, *n* = 34) and VV2 (156 ng/mL, *n* = 12) (*p* = 0.32) (Fig. 2b). To assess the potential alteration of plasma YKL-40 along disease progression, CJD cases were stratified in three groups according to the stage of the disease where the blood was collected (1/early: *n* = 13, 2/middle: *n* = 16 and 3/late: *n* = 40 disease stage). We found a significant increase of YKL between late disease stage (215 ng/mL) and early disease stage (104 ng/mL, *p* = 0.036) (Fig. 2c). However, lack of association between YKL-40 concentrations and disease duration was present in the CJD group (*p* = 0.32) (Fig. 2d).

## Discussion

Many efforts are devoted to discover blood-based clinical biomarkers for the diagnosis of neurodegenerative diseases. In this search, plasma YKL-40 has been positioned as a promising candidate and a significant elevation was reported in mild AD-type dementia and early AD compared to controls [7, 18], but the significance of these findings in the differential diagnostic context of neurodegenerative dementias was unknown. Although we were able to detect increased mean plasma YKL-40 concentrations in AD compared to controls, the comparative analysis in different dementia conditions indicated that only statistically significant alterations were observed between LBD and HC and between CJD and HC, ND, AD and VaD. Resultant AUC values were low (< 0.8), except from that obtained between CJD and HC, which reached 0.81. However, this value was still far from that rendered by CSF YKL-40 between CJD and neurological controls (AUC = 0.92) [4].

Plasma YKL-40 levels were influenced by age but not by sex, as it is the case of CSF YKL-40 [7]. Within the CJD group, no differences in YKL-40 levels were detected based on the most prevalent clinical subtypes, MM1 and VV2, even though they display different clinic-pathological outcomes. Similarly, no association between plasma YKL-40 and codon 129 polymorphism of *PRNP* either disease duration was observed, in agreement with the data obtained for CSF YKL-40 [4]. Thus, the moderate AUC value achieved in the discrimination of CJD from HC presents robustness in front of clinical heterogeneity. By contrast, we found that CJD cases at early disease stages present significantly lower values than those at late stage. Therefore, although the value of plasma YKL-40 as diagnostic biomarker is rather limited and our data do not support its usefulness in the differential diagnostic context, this marker may serve in the evaluation of disease progression and monitoring of potential therapeutic interventions.

Being CSF YKL-40 considered a sound marker of neuro-inflammation, its rise in CJD, AD and other neurodegenerative diseases characterized by neuro-inflammatory profile is expected [4–6, 19]. However, the weak correlation between CSF and plasma YKL-40 previously reported [7, 20] and the fact that YKL-40 levels in blood are not altered in diagnostic groups where CSF levels are so [20] indicates that alternative mechanisms, other than direct CSF-blood exchange, might exist to explain the regulation of plasma YKL-40 herein observed. In the brain, the expression of YKL-40 upon inflammation conditions is mainly attributed to reactive astrocytes [7, 21–23]. In the case of CJD, the presence of perivascular astrocytes was detected in cortical regions [4]. Thus, it is tempting to speculate that, upon damage of the brain blood vessels or impairment of the blood-brain barrier, which is common in many neurodegenerative diseases [24], this subset of astrocytes could release brain-derived YKL-40 in the blood. In this case, the increase of YKL-40 in plasma at advanced CJD stage that we observed may reflect, not only the degree of neuro-inflammation, but also suspected damage in brain blood vessels. Further investigations will be necessary to demonstrate this hypothesis and clarify the origin of YKL-40 in the plasma of patients with neurodegenerative dementia.

## Conclusions

Altogether, our study indicates that plasma YKL-40 may contribute to the diagnosis of CJD regardless of clinical heterogeneity, but it should be combined with other blood-based biomarkers to increase its diagnostic performance. Contrarily, our data do not support the use of this marker in the challenging differential diagnostic context of neurodegenerative dementias. Additionally, a potential use as a CJD progression marker is envisaged.

## Data Availability

The datasets used during the current study are available from the corresponding authors on reasonable request.

## Abbreviations

AD: Alzheimer’s disease
AUC: Area under the curve
CJD: Creutzfeldt-Jakob disease
CSF: Cerebrospinal fluid
FTD: Frontotemporal dementia
HC: Healthy controls
LBD: Lewy body dementia
ND: Neurological disease controls
VaD: Vascular dementia
